# The risk factors for benign small bowel obstruction following curative resection in patients with rectal cancer

**DOI:** 10.1186/s12957-018-1510-7

**Published:** 2018-10-22

**Authors:** Liang Tang, Peng Zhao, Dalu Kong

**Affiliations:** Department of Colorectal Cancer, Tianjin Medical University Cancer Institute and Hospital, Key Laboratory of Cancer Prevention and Therapy, National Clinical Research Center of Cancer, Huanhuxi Road, Hexi District, Tianjin, People’s Republic of China

**Keywords:** Risk factor, Small bowel obstruction (SBO), Rectal cancer, Open surgery, Radiotherapy, Resection

## Abstract

**Background:**

So far there have been limited studies about the risk factors for benign small bowel obstruction (SBO) after colorectal cancer surgery. This study aimed to determine the factors affecting the development of benign SBO following curative resection in patients with rectal cancer.

**Methods:**

Patients (3472) receiving curative resection of rectal cancer at the Department of Colorectal Cancer, Tianjin Medical University Cancer Institute and Hospital, between January 2003 and December 2012 were retrospectively studied. The incidence of benign SBO and its risk factors were then determined.

**Results:**

The incidence of benign SBO was 7.3% (253/3472) in follow-up studies with an average time of 68 months. Further, 27% (68/253) of the patients received operative treatment because of the signs of strangulation or the lack of clinical improvement with conservative management. Open surgery and radiotherapy were defined as the risk factors for benign SBO after curative resection in patients with rectal cancer (*P* <  0.001).

**Conclusion:**

Open surgery plus radiotherapy led to an increased risk of benign SBO in rectal cancer patients receiving curative resection.

## Background

Previously, abdominal surgery is the leading cause of adhesive small bowel obstruction (SBO) [1, 2]. Patients receiving colorectal surgeries are at higher risk of postoperative SBO, which might be resulting from the dissection in the peritoneal cavity [3–5]. It has been shown that the incidence of SBO requiring hospitalization following colorectal resection was 3.6% 3 years after surgery [6] and that SBO was found in ~ 9% patients with colorectal procedures [7]. However, the said findings were obtained based on information from various colorectal surgeries, such as anorectal procedures, or different diseases like carcinomatosis. Therefore, these findings cannot demonstrate the accurate incidence of benign SBO following curative resection in rectal cancer patients.

To date, there have been limited studies about the risk factors of benign SBO following colorectal cancer surgery [6, 8–12]. In the present study, we determined the incidence of benign SBO and its risk factors following curative resection for rectal cancer, providing guidance for operative treatment of rectal cancer patients.

## Methods

### Patients and the diagnosis of benign SBO

This retrospective research was approved by the Ethics Committee of Tianjin Medical University Cancer Institute and Hospital. Written informed consent for publication of the patient’s information was obtained from all patients.

In total, 3472 consecutive patients undergoing rectal cancer surgery at the Department of Colorectal Cancer, Tianjin Medical University Cancer Institute and Hospital, between January 2003 and December 2012 were enrolled. The clinicopathological data were extracted from patient files. The patients’ information was collected, such as age, gender, type of primary surgery, surgery duration, and hospitalized days. The patients were followed up with an average time of 68 (7–89) months.

The diagnosis criteria of benign SBO were as follows: patients showing clinical symptoms as below, including ventosity, constipation, colicky abdominal pain, nausea, and hyperactive bowel sounds. The patients with SBO were further confirmed when fluid levels in dilated loops were shown by the plain abdominal X-ray. The contents of serum carcinoembryonic antigen in patients were also measured. Besides, the possible tumor recurrence or carcinomatosis in patients was determined by the imaging procedures of computed tomography (CT) and positron emission tomography (PET).

The exclusion criteria were as follows: patients with distant metastasis (e.g., liver, lung, brain, or peritoneal carcinomatosis), patients died 30 days after surgery, patients with SBO resulting from cancer recurrence or peritoneal carcinomatosis, and patients without complete follow-up data.

### Statistical analysis

The SPSS 13.0 software (SPSS, Chicago, IL, USA) was used for statistical analysis. The correlation of benign SBO and clinicopathological factors was evaluated by a chi-square (*χ*^*2*^) test or Fisher’s exact test. Risk factors for benign SBO following curative resection in rectal cancer patients were analyzed by univariate and multivariate logistic regression analysis. *P* <  0.05 was taken as statistically significant.

## Results

Two hundred and fifty-three patients (7.3%, 253/3472) hospitalized were diagnosed with benign SBO. Among these patients, 247 cases (97.6%, 247/253) were first subjected to conservative treatment. The conservative treatment included venous transfusion, total parenteral nutrition, gastric tube insertion, coloclysis, and bowel rest. However, conservative treatment was ineffective for 95 patients, who were then subjected to surgeries. Additionally, six (2.4%) patients underwent laparotomy in the initial 12 h of hospitalization due to the possibility of small bowel strangulation indicated by imaging.

The categories of surgery included lysis of adhesions, small bowel resection, ileocecectomy, and colectomy. When comparing to the conservative treatment group, surgical treatment group possessed a lower incidence of recurrent benign SBO, higher mortality, and longer hospital stay (Table 1). It was noteworthy that most patients undergoing operation received primary conservative treatment; therefore, a careful determination of hospitalized days should be taken.

**Table 1:** The outcome of patients with small bowel obstruction (SBO) through different treatments (*N* = 253)

	Conservative treatment group (*n* = 152)	Surgical treatment group (*n* = 101)	*P* value
SBO recurrence	54 (35.5%)	15 (14.8%)	< 0.001
Mean hospital stay (days)	5.8	14.5	< 0.001
Mortality	2 (1.32%)	4 (3.96%)	0.176

As shown in Table 2, multiple variables were likely correlated with benign SBO, including surgical approach, radiotherapy, antiadhesive materials, pelvic peritoneum sutured, blood loss, and tumor stage (TNM). Then the multivariate logistic regression analysis demonstrated that open surgery (OR, 8.25; 95% CI, 2.18–17.32; *P* <  0.001) and radiotherapy (OR, 6.13; 95% CI, 1.47–15.36; *P* <  0.001) were the independent risk factors of benign SBO in rectal cancer patients receiving curative resection (Table 3).

**Table 2:** Risk factors for benign small bowel obstruction (SBO) by the univariate analysis

Variables	No. of patients	No. of SBO	OR	95% CI	*P* value
Age			0.82	0.62–1.07	0.14
≤ 60	1318	85			
> 60	2154	168			
Gender			0.87	0.67–1.12	0.267
Male	1805	123			
Female	1667	130			
Type of surgery			NA	NA	0.299
Anterior resection	2547	176			
Abdominoperineal resection	783	67			
Hartmann’s operation	142	10			
Surgical approach			1.59	1.21–2.09	0.001
Open	2014	172			
Laparoscopic	1458	81			
Duration of surgery			0.78	0.59–1.01	0.062
≤ 3 h	2378	160			
> 3 h	1094	93			
Radiotherapy			2.49	1.92–3.23	< 0.001
Yes	901	113			
No	2571	140			
Chemotherapy			0.81	0.63–1.07	0.144
Yes	2341	160			
No	1131	93			
Antiadhesive materials			1.42	1.05–1.93	0.023
Yes	2489	197			
No	983	56			
Pelvic peritoneum sutured			1.48	1.12–1.96	0.007
Yes	2638	174			
No	834	79			
Blood loss			0.71	0.54–0.93	0.012
≤ 400	2513	166			
> 400	959	87			
Previous laparotomy			0.75	0.35–1.62	0.598
Yes	125	7			
No	3347	246			
Tumor stage (TNM)			NA	NA	0.043
I	512	35			
II	1295	78			
III	1665	140

**Table 3:** Independent risk factors for benign small bowel obstruction (SBO) by logistic regression analysis

Variables	χ^2^	*P* value	OR	95% CI
Open surgery	15.07	< 0.001	8.25	2.18–17.32
Radiotherapy	11.69	< 0.001	6.13	1.47–15.36

## Discussion

SBO is a potentially life-threatening complication after primary rectal cancer surgery. Though the patients undergoing colorectal surgery are at high risk of SBO [3, 5], there have been limited studies about the accurate incidence of SBO following colorectal operation [4, 6, 7, 13–16]. It has been reported that the occurrence rate of SBO following colorectal resection varied between 1.5–12.5% [7, 15] and 24–32.6% [6, 14, 16]. In this study, the incidence of benign SBO following rectal cancer resection was 7.3% (253/3472), which was lower compared with previous studies [6, 7, 14–16]. This difference might be resulted from the technological innovation, especially after the wide application of laparoscopic surgery [6]. The incidence of adhesive SBO was shown to be 32.6% in a 10-year follow-up study [16]. However, the average follow-up period in our study was 68 months, possibly leading to the lower benign SBO incidence. It has been found that the average period between primary colorectal operation and SBO was 8.4 years [17]. Therefore, a long-range follow-up is required for determining the accurate incidence of SBO in rectal cancer patients receiving initial operation.

In our study, the results showed that open surgery was a risk factor for benign SBO following rectal cancer operation, concurring with a previous finding, increased incidence of SBO after laparotomy [18]. Studies have shown that the abdominal wall damage and intestinal operation increased inflammatory reactions [19], possibly resulting in the occurrence of SBO after an open rectectomy [8]. In contrast, multiple studies have demonstrated that laparoscopic proctectomy could reduce adhesion-related complications and produce better short-term outcome [20–22]. Besides, in this study, the incidence of benign SBO following rectal cancer surgery in the laparoscopic group was much lower compared with the open surgical group. Taken together, the results demonstrated the advantages of laparoscopy, providing a theoretical basis for treating rectal cancer patients using laparoscopic surgery.

Owing to the advantages of adjuvant radiotherapy, an increasing number of rectal cancer patients choose this option. But the long-range effects of radiotherapy should be completely studied to avoid the undesired side effects. It is well established that bowel obstruction is a long-term post-irradiation complication, and the incidence of bowel obstruction is increased when large quantities of small bowel are exposed to irradiation, especially the doses more than 50–55 Gy [23]. In the long term, small bowel, affected by heavy quantities of irradiation could develop fibrosis and ischemia, might show as SBO. However, the incidence of post-irradiation SBO varies a lot, and no definite reasons could account for this variation. The fixed loops of small bowel in pelvis were observed in 65% of patients receiving postoperative irradiation relative to 18% of patients without operation [24]. As fixed bowel maintains location during the treatment, the fixed bowel is potentially exposed to a significantly higher amount of irradiation compared with mobile bowel. Therefore, caution should be taken in the application of radiotherapy. A previous study showed that no difference in the occurrence rate of SBO was observed between surgery group (11%) and surgery combined with short-term preoperative irradiation group (11%) during a 5.1-year follow-up, suggesting the superiority of short-term preoperative irradiation over postoperative irradiation [25]. However, in our study, most patients (683/901) undergoing radiotherapy were treated with postoperative irradiation. And we found that radiotherapy was the risk factor for SBO in rectal cancer patients subjected to radical resection, which was in line with previous findings [9]. Taken together, these results reinforced the superiority of preoperative radiotherapy to reduce the long-run risk of developing SBO.

Notably, a recent study examined the risk factors for SBO that occurred within 30 days following anterior resection for rectal cancer [10]. Patients with perioperative complications other than SBO and with simultaneous resection of other organs were excluded from the study. The univariate logistic regression was conducted to screen for the factors related to the occurrence of SBO; the identified factors were then used in a multivariate logistic model to evaluate the independent risk factors for SBO; they found D3 node dissection and defunctioning ileostomy formation were the independent risk factors for early postoperative SBO after anterior resection for rectal cancer. Therefore, basically, their research and our research are methodologically consistent; the biggest difference lies in the subjects of interest. The subjects with a long-term follow-up (68 months) in our study might result in the finding of different risk factors for SBO from previous studies.

## Conclusion

In conclusion, we found that open surgery and radiotherapy were the independent risk factors of benign SBO following curative resection in rectal cancer patients. Therefore, laparoscopic surgery was confirmed to be a useful countermeasure against the long-term risk of benign SBO.
